# Composite cuticle with heterogeneous layers in the leaf epidermis of *Ficus elastica*

**DOI:** 10.1186/s42649-019-0022-4

**Published:** 2019-12-24

**Authors:** Ki Woo Kim

**Affiliations:** 1 School of Ecology and Environmental System, Kyungpook National University, Sangju, 37224 Republic of Korea; 2 Tree Diagnostic Center, Kyungpook National University, Sangju, 37224 Republic of Korea

**Keywords:** Cuticle, Cutin, Epidermis, Polysaccharide, Wax

## Abstract

Two distinct layers in terms of texture and electron density were observed in the leaf cuticle of *Ficus elastica* using transmission electron microscopy. As depicted in a model, an inner polysaccharide-rich layer and an outer cutin (or cutan)-rich layer may support the composite, heterogeneous concept of the leaf cuticle.

The cuticle represents the outermost surface structure of a variety of organisms such as plants and insects. As the interface between plants and their environment, the plant cuticle plays a number of roles mostly associated with protection against biotic and abiotic stresses including pathogen infection and water loss (Domínguez et al., 2017). It usually consists of an insoluble polyester cutin and the waxes (Kim, 2012). Originated in the nineteenth century, the concept of the plant cuticle has been challenged and revised through the accumulation of different leaf cuticle structures (Fernández et al., 2016). The early view of the cuticle as a cellulose-free region has been changed to the prevailing concept of the cuticle as a chemically and structurally heterogeneous region (Fernández et al., 2016). However, there is a severe imbalance between the number of plant species observed at the cuticle level using transmission electron microscopy and the number of extant plant species (Domínguez et al., 2017). Structural variations can be found even within the same species and within a cuticle section for transmission electron microscopy (Fernández et al., 2016).

Leaves of the rubber tree *Ficus elastica* were fixed, dehydrated, and embedded in LR white resin (Kim, 2008). Energy-filtering transmission electron microscopy revealed the transverse section of the leaf epidermis (Fig. 1). Two different layers in terms of texture and electron density were observed in the leaf cuticle on the epidermal cell wall: (i) an inner electron-translucent layer and (ii) an outer electron-dense layer below the epicuticular wax layer. The inner electron-translucent region possessed cellulose fibrils in the matrix, probably derived from the underlying epidermal cell wall.

**Fig. 1:**
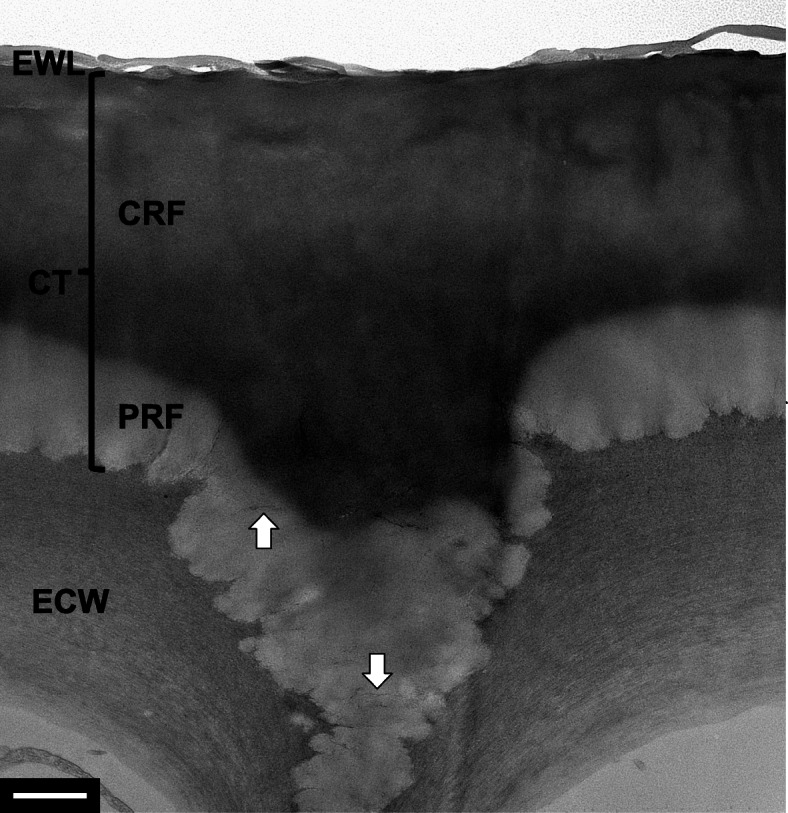
Transmission electron micrograph of the heterogeneous cuticle in the leaf epidermis of *Ficus elastica*. A polysaccharide-rich fraction and a cutin-rich fraction constituted the cuticle. Arrows = cellulose fibrils. CRF = cutin-rich fraction. CT = cuticle. ECW = epidermal cell wall. EWL = epicuticular wax layer. PRF = polysaccharide-rich fraction. Bar = 1 μm

The heterogeneous cuticle structure was strikingly similar to that of the model depicted by Heredia-Guerrero et al. (2014). They proposed the composite nature of the leaf cuticle having a polysaccharide-rich fraction and a cutin -rich fraction. In addition, the result from this study was consistent with that on the same tree species by others (Guzmán-Delgado et al., 2016). They verified the presence of cutan, an alternative form to cutin, in the leaf cuticle using infrared spectroscopy and electron microscopy. While the previous article showed regions or islands with heterogeneous structures in the leaf cuticle, this study demonstrated two distinct layers in cuticle structure. Taken together, the inner and outer layers in the leaf cuticle may correspond to a polysaccharide-rich fraction and a cutin (or cutan)-rich fraction, respectively (Heredia-Guerrero et al. 2014). These results support the composite, heterogeneous concept of the leaf cuticle. Natural composite biopolymers like the plant cuticle can provide insights on the design and development of bioplastics with a high biodegradability and nontoxicity (Domínguez et al., 2017).

## Data Availability

Data and materials available on request.
